# Supplementary material for: Modeling glioblastoma heterogeneity as a dynamic network of cell states
Source: Mol Syst Biol. 2021 Sep 16;17(9):e10105. doi: 10.15252/msb.202010105 (PMC8444284; doi:10.15252/msb.202010105)
Supplement: Supplementary file 6 — Source Data for Figure 5 [file MSB-17-e10105-s004.zip › Figure5A_sourcedata/GSEA_3071/hallmarks_stateVI.GseaPreranked.1623742557031/HALLMARK_TGF_BETA_SIGNALING.html]

Details for gene set HALLMARK\_TGF\_BETA\_SIGNALING[GSEA]

|  || Dataset | state63071 |
| Phenotype | NoPhenotypeAvailable |
| Upregulated in class | na\_pos |
| GeneSet | HALLMARK\_TGF\_BETA\_SIGNALING |
| Enrichment Score (ES) | 0.28931168 |
| Normalized Enrichment Score (NES) | 1.4755629 |
| Nominal p-value | 0.07826087 |
| FDR q-value | 0.09294076 |
| FWER p-Value | 0.39 |
Table: GSEA Results Summary

  

Fig 1: Enrichment plot: HALLMARK\_TGF\_BETA\_SIGNALING      
 Profile of the Running ES Score & Positions of GeneSet Members on the Rank Ordered List

  

| PROBE | GENE SYMBOL | GENE\_TITLE | RANK IN GENE LIST | RANK METRIC SCORE | RUNNING ES | CORE ENRICHMENT || 1 | SERPINE1 |  |  | 0 | 1.673 | 0.1008 | Yes |
| 2 | THBS1 |  |  | 12 | 1.097 | 0.1639 | Yes |
| 3 | SMURF2 |  |  | 35 | 0.930 | 0.2139 | Yes |
| 4 | NOG |  |  | 77 | 0.793 | 0.2504 | Yes |
| 5 | PPP1R15A |  |  | 98 | 0.738 | 0.2893 | Yes |
| 6 | KLF10 |  |  | 506 | 0.453 | 0.2039 | No |
| 7 | BMP2 |  |  | 940 | 0.354 | 0.1053 | No |
| 8 | FKBP1A |  |  | 1118 | 0.327 | 0.0760 | No |
| 9 | UBE2D3 |  |  | 1361 | 0.296 | 0.0268 | No |
| 10 | PPP1CA |  |  | 1525 | 0.276 | -0.0017 | No |
| 11 | FURIN |  |  | 1702 | 0.260 | -0.0348 | No |
| 12 | ID3 |  |  | 1835 | -0.254 | -0.0560 | No |
| 13 | TRIM33 |  |  | 2086 | -0.293 | -0.1076 | No |
| 14 | NCOR2 |  |  | 2134 | -0.303 | -0.1024 | No |
| 15 | SKI |  |  | 2221 | -0.318 | -0.1070 | No |
| 16 | ARID4B |  |  | 2282 | -0.330 | -0.1038 | No |
| 17 | APC |  |  | 2301 | -0.333 | -0.0887 | No |
| 18 | BMPR1A |  |  | 2555 | -0.389 | -0.1354 | No |
| 19 | RAB31 |  |  | 2806 | -0.459 | -0.1769 | No |
| 20 | SMAD1 |  |  | 2815 | -0.461 | -0.1513 | No |
| 21 | CTNNB1 |  |  | 2863 | -0.483 | -0.1353 | No |
| 22 | BMPR2 |  |  | 2930 | -0.506 | -0.1231 | No |
| 23 | TGFBR1 |  |  | 3017 | -0.548 | -0.1139 | No |
| 24 | ID2 |  |  | 3026 | -0.554 | -0.0827 | No |
| 25 | SKIL |  |  | 3188 | -0.652 | -0.0880 | No |
| 26 | SPTBN1 |  |  | 3197 | -0.661 | -0.0504 | No |
| 27 | PMEPA1 |  |  | 3338 | -0.804 | -0.0407 | No |
| 28 | TGIF1 |  |  | 3380 | -0.860 | -0.0002 | No |
| 29 | WWTR1 |  |  | 3519 | -1.185 | 0.0330 | No |
Table: GSEA details [plain text format]

  

Fig 2: HALLMARK\_TGF\_BETA\_SIGNALING: Random ES distribution      
 Gene set null distribution of ES for **HALLMARK\_TGF\_BETA\_SIGNALING**
